# Two-dimensional wide-bandgap GeSe_2_ vertical ultraviolet photodetectors with high responsivity and ultrafast response speed†

**DOI:** 10.1039/d2na00565d

**Published:** 2022-10-03

**Authors:** Yong Yan, Jie Li, Shasha Li, Mengna Wang, Gaoli Luo, Xiaohui Song, Suicai Zhang, Yurong Jiang, Ruiping Qin, Congxin Xia

**Affiliations:** School of Physics, Henan Normal University Xinxiang Henan province China yanyong@htu.edu.cn xiacongxin@htu.edu.cn

## Abstract

Germanium selenide (GeSe_2_), as a typical member of 2D wide bandgap semiconductors (WBSs), shows great potential in ultraviolet (UV) optoelectronics due to its excellent flexibility, superior environmental stability, competitive UV absorption coefficient, and significant spectral selectivity. However, the GeSe_2_-based UV photodetector suffers from high operation voltages and low photocurrent, which prevents its practical imaging applications. In this work, we report an elevated photocurrent generation in a vertical stacking graphene/GeSe_2_/graphene heterostructure with low operation voltage and low power consumption. Efficient collection of photoexcited carriers in GeSe_2_ through graphene electrodes results in outstanding UV detection properties, including a pronounced responsivity of 37.1 A W^−1^, a specific detectivity of 8.83 × 10^11^ Jones, and an ultrahigh on/off ratio (∼10^5^) at 355 nm. In addition, building a Schottky barrier between GeSe_2_ and graphene and reducing the channel length can increase the photoresponse speed to ∼300 μs. These accomplishments set the stage for future optoelectronic applications of vertical 2D WBS heterostructure UV photodetectors.

## Abstract 2

Germanium selenide (GeSe_2_), as a typical member of 2D wide bandgap semiconductors (WBSs), shows great potential in ultraviolet (UV) optoelectronics due to its excellent flexibility, superior environmental stability, competitive UV absorption coefficient, and significant spectral selectivity.

## Graphical abstract

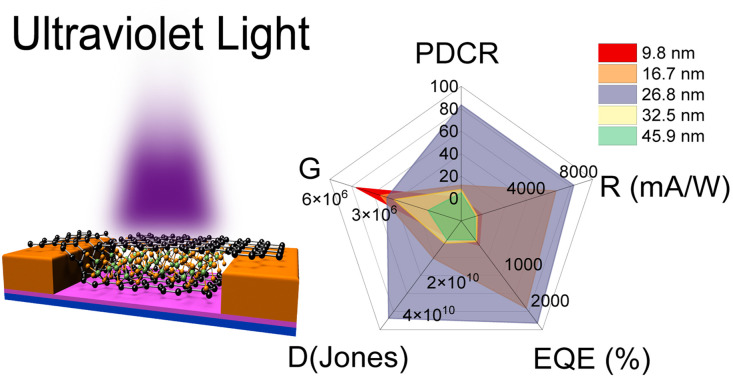

## Introduction

Advanced ultraviolet (UV) photodetectors that convert UV radiation signals into electronic signals are of great significance because they have extensive applications in the fields of military warning, spatial communication, ozone hole monitoring, and so on.^1^ In the past decades, wide-bandgap semiconductors (WBGSs), such as ZnO, TiO_2_, Ga_2_O_3_, and SnO_2_, have been used as excellent building blocks for UV optoelectronic devices with good responsivity, high specific detectivity, and low noise characteristics.^2–6^ However, the oxygen-related hole-trapping state induces a low response speed and poor anti-disturb ability, owing to the large density of defects and dangling bonds at the surface.^7^ Recently, 2D wide-bandgap semiconductors (2D WBGSs) have attracted significant attention because of their dangling-bond-free surfaces.^8^ For example, Ga_2_In_4_S_9_,^9^ NiPS_3_,^10^ GaPS_4_,^11^ and BiOBr^12^ are potential candidate materials for UV photodetectors. However, the synthesis is usually difficult and expensive owing to the complex chemical composition and structural polymorphism of these materials. It is known that elemental or binary compounds can be a more efficient choice for the preparation of detectors. For instance, hexagonal boron nitride (h-BN) with a direct wide bandgap of 5.9 eV, large absorption coefficient (7.5 × 10^5^ cm^−1^), superior chemical inertness, shows a promising role in UV photodetection.^12,13^

2D WBGS germanium diselenide, GeSe_2_, attracts considerable attention due to its excellent anisotropic properties, high stability, strong nonlinearity, and fast response.^14^ Moreover, single layer GeSe_2_ has [GeSe_4_] tetrahedrons as the basic building blocks. The tetrahedrons form [GeSe_4_]_*n*_ chains by corner-sharing along the *a*-axis, which are connected by [Ge_2_Se_8_] double tetrahedra by edge-sharing along the *b*-axis, forming an in-plane anisotropic geometry.^15^ Zhou *et al.* experimentally verified strong in-plane anisotropic properties of the rhombic GeSe_2_ layer.^14^ Additionally, Yang *et al.* demonstrated the weak interlayer interaction in GeSe_2_.^16^ It is well known that the electronic and optical properties of 2D materials are dependent on the layer number from multilayer to monolayer. Recently, exfoliated monolayer GeSe_2_ nanosheets have been found to have a direct bandgap of 2.96 eV.^17^ Meanwhile, GeSe_2_ is a well-defined p-type semiconductor with a hole mobility of 690 cm^2^ V^−1^ s^−1^, which is higher than that of most 2D semiconductors.^18^ However, the GeSe_2_ photodetector shows an ultralow photocurrent (∼pA) potentially retarding its practical application in UV detection, which is still required to be comprehensively improved.

In this study, we constructed a vertical GeSe_2_-based heterostructure with graphene (labeled as Gr) electrodes for high-performance UV photodetection. We systematically discussed the carrier transport mechanism in Gr/GeSe_2_/Gr devices, including direct tunneling (DT) to thermionic emission (TE) transport. The redistribution of space charge is increased by the external electric field, which improves the carrier collection efficiency. Moreover, the graphene electrode enhances the carrier extraction, resulting in an excellent photoresponse with a high responsivity of 7.6 × 10^3^ mA W^−1^, a detectivity of 5.0 × 10^10^ Jones, a large on/off ratio (∼10^5^), and a fast response speed of ≈0.3 ms under 405 nm laser irradiation. These results provide a scientific approach that directs a valuable way to prepare high-performance UV photodetectors.

## Experimental details

### Transfer method and device fabrication

Bulk GeSe_2_ crystals were synthesized by a chemical vapor transport method.^17^ The 2D GeSe_2_ flakes were achieved by mechanical exfoliation from bulk crystals with a polydimethylsiloxane (PDMS, Shanghai Onway Technology Co. Ltd) stamp-assisted peeling method.^11^ High-quality 90 nm SiO_2_/p^+^-Si wafer was used as the substrate. For better material adhesion, the substrate was placed on the micro heater of the transfer platform during the transfer process, and after aligning and placing the graphene in the center of the substrate and the temperature was ramped up to 80 °C, the PDMS stamp was allowed to adhere to the SiO_2_ surface. The temperature was then cooled down to 40 °C to release the glass slide and PDMS stamp from the SiO_2_/p^+^-Si substrate, leaving only the graphene flakes on top of the substrate. Following the same transfer procedure, the exfoliated GeSe_2_, and graphene were stacked layer by layer forming a Gr/GeSe_2_/Gr vertical vdW heterostructure. Finally, the devices were annealed at 150 °C for 3 h in a vacuum chamber. Standard photolithography was employed to define the source and drain electrode patterns on the wafer, and a 40 nm Au film was evaporated as the contact metal.

### Characterization and measurements

The thickness of 2D nanosheets was identified by optical microscopy and atomic force microscopy (AFM, Dimension Icon, Bruker) in a tapping mode. The Raman spectrum was measured by confocal Raman microscopy (Horiba, Evolution HR, 532 nm laser excitation at room temperature). The elemental analysis was proceeded using SEM (SUPRA40, Carl Zeiss AG) combined with energy-dispersive spectrometry (EDS). The electronic and photoelectronic measurements were performed on a probe station (450PM, Micromanipulator) equipped with a semiconductor characterization system (B1500A, Keysight).

## Results and discussion

**Fig. 1:**
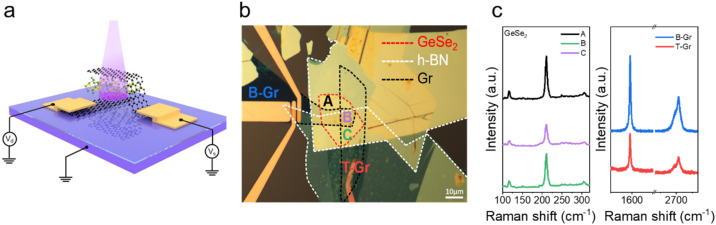
Schematic illustration (a) and optical micrograph (b) of the device with a multilayer GeSe_2_ semiconductor sandwiched between the T-Gr and B-Gr electrodes. (c) Raman spectra were collected from points A, B, C, bottom Gr, and top Gr, respectively.

Fig. 1a shows the schematic diagram of the Gr/GeSe_2_/Gr FET device, in which a GeSe_2_ layer is sandwiched between two cross-stacked metallic Gr layers on a 90 nm SiO_2_/p^+^-Si wafer. The 2D layers were mechanically exfoliated from GeSe_2_ and graphite crystals using the polydimethylsiloxane (PDMS) tape. The top and bottom graphene layers serve as the source and drain electrodes, respectively. The device was encapsulated with hexagonal boron nitride (hBN) layers, avoiding carrier scattering caused by adsorbents and impurities between the vertical heterostructure and insulating the device from the air.^19^ The overlapping area is 16.8 × 7.2 μm^2^ and the thickness (*d*) of the GeSe_2_ active layer is 70.3 nm. The Raman spectra in Fig. 1c were taken from points A, B, and C, respectively. Three Raman peaks at 95, 116, and 210 cm^−1^ correspond to the *A*_g_ typical vibrational modes of β-GeSe_2_.^20,21^ There is no detectable change in the GeSe_2_ peak position at different positions, indicating no mutual stress effect at the van der Waals interface. The G (1583 cm^−1^) and 2D (2748 cm^−1^) peaks were observed at the bottom Gr (9.8 nm, blue line) and the top Gr (4.3 nm, red line) layers, respectively.^22^ In addition, the detected stoichiometric ratio of Ge and Se is about 1 : 2 (Fig. S1†). These results confirmed we prepared a high-quality Gr/GeSe_2_/Gr device by the dry transfer process.

**Fig. 2:**
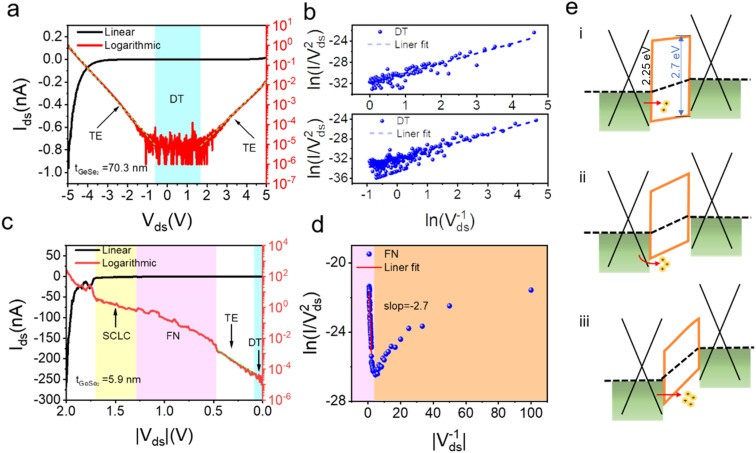
(a) *I*–*V* characteristics of the thick GeSe_2_ (*d* = 70.3 nm) device, the black and red lines are in linear and logarithmic coordinates, respectively. (b) ln(*I*/*V*^2^) *vs.* ln(*V*_ds_^−1^) plot for the DT behavior. The top panel shows the tunneling current when the drain bias is between −0.5 and 0 V, and the bottom panel shows the tunneling current when the drain bias is between 0 and 1.5 V. (c) *I*–*V* characteristics of the GeSe_2_ (*d* = 5.9 nm) device. (d) ln(*I*/*V*^2^) *vs.* 1/*V* plot (Fowler–Nordheim tunneling plot) when the drain bias is lower than −0.45 V. (e) Schematic illustration of the band diagrams.

We first investigated the current–voltage (*I*–*V*) characteristics of the GeSe_2_ device with the drain bias voltage sweeping from 0 to ±5 V. The thickness of the GeSe_2_ layer was about 70.3 nm (Fig. S2†). A Schottky contact was formed between semi-metallic Gr and 2D GeSe_2_ (Fig. 2a), which is similar to the contact between Gr and GaN.^23^ There are three ways for the charge to be transported through the Schottky barrier: DT, TE, and FN mechanisms.^19,24–28^ When −0.5 V < *V*_ds_ <1.5 V, the current (at ∼pA) satisfies the DT mechanism. The carrier transport schematic is shown in Fig. 2e(i). Electrons cannot move across the barrier and only a few carriers can pass through the DT process, generating a small current. The direct tunneling process can be described by^19,29^1
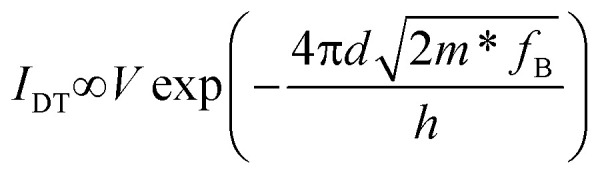
2
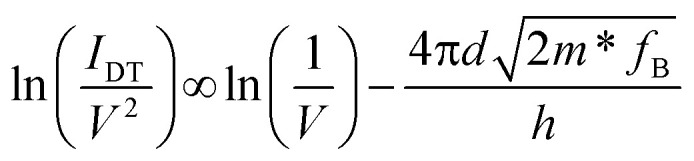
where *d* is the tunneling barrier thickness and *f*_B_ is the tunneling barrier. Fig. 2a shows the linear ln(1/*V*) − ln(*I*/*V*^2^) curve, which is in agreement with the DT mechanism. When *V*_ds_ > 1.5 V or *V*_ds_ < −0.5 V, in Fig. 2a, the ln *I*–*V* curve exhibits a linear shape (Fig. S2†), indicating that charge carriers follow the TE mechanism. The TE mechanism shows the relationship between the current and voltage as shown by^30^3
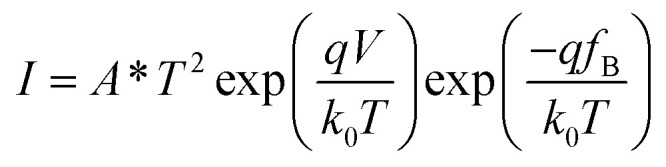
4
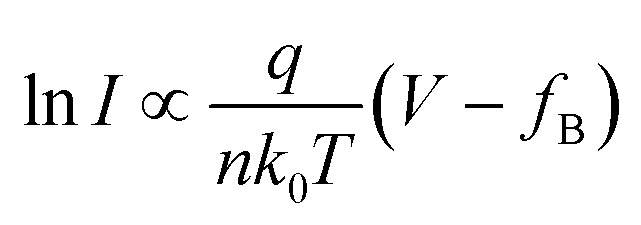
where A* is the effective Richardson's constant (A* = 4πm*qk_0_^2^/*h*^3^, m* is the effective carrier mass), *q* is the unit electron charge, *k*_0_ is the Boltzmann constant, *T* is temperature, *f*_B_ is the effective barrier height, and *V* is the bias voltage. *n* is the ideality factor. The slope is equal to *e*/*nkT* and allows one to extract the ideality factor *n* using5
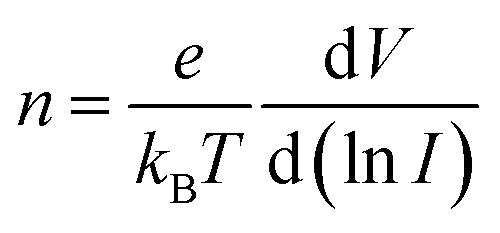


Thus, the ideality factor *n* is 14.3. According to the Shockley–Read–Hall (SRH) recombination theory, the ideality factor of the recombination current is predicted to be *n* = 2 or less, which assumes recombination *via* isolated point defect levels. The larger ideality factor suggests other recombination currents crossing this Schottky junction.

As the physical properties of 2D materials are sensitive to thickness, we further explored the electrical performance of the device with thin GeSe_2_ (5.9 nm). When 0 V < *V*_ds_ < 0.5 V, similar to the thick devices also have DT and TE processes (Fig. S2†). For larger voltage biases (>0.5 V), FN tunneling is expected to dominate the charge transport. In the FN tunneling mechanism, the tunneling potential is modulated into a triangle barrier and the tunneling current can be described as^19^6
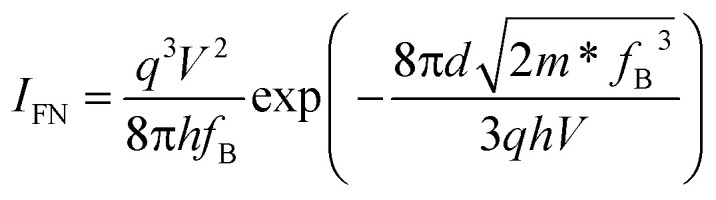
7
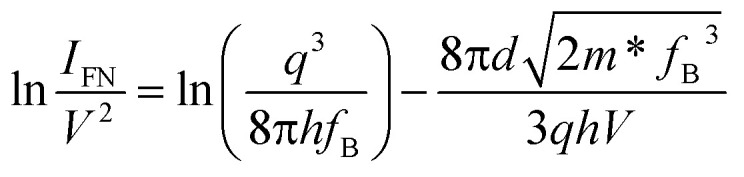


In Fig. 2d, the ln(*I*/*V*^2^) − 1/*V* curve is linear with a slope 
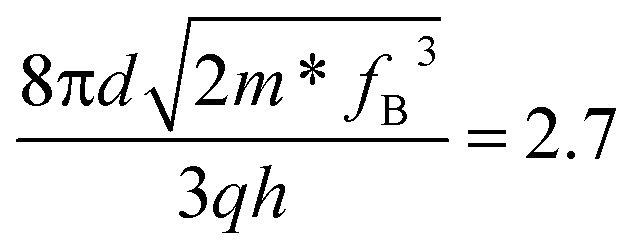
. The effective mass of the hole in the GeSe_2_ tunneling barrier can be extracted by its band structure fitting as m* = 1.8*m*_0_.^20^ The tunneling barrier height *f*_B_ can be estimated to be 0.45 eV, confirming the FN tunneling behavior of the hole. The band alignment of Gr/GeSe_2_ heterostructures shows that the conduction and valence band offsets are 2.25 eV and 0.45 eV, respectively. (Fig. S3†) With the large bias voltage, the energy band renders very strong bending, leading to a shift in transport mechanisms, in which holes can inject into the GeSe_2_ valence bands.

**Fig. 3:**
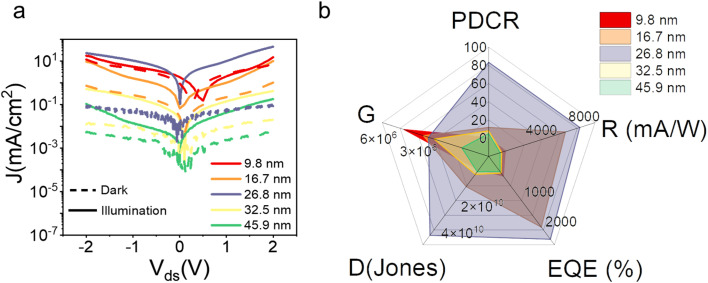
The optoelectronic performances of the GeSe_2_ devices with different thicknesses. (a) *J*–*V* logarithm plots for the dark state (dashed line) and 405 laser irradiation (solid line). (b) PDCR, responsivity (*R*), External Quantum Efficiency (EQE), detectivity (*D*), gain (*G*) extracted from (a).

The optoelectronic properties of the device were then systematically studied to examine its potential application in UV photodetection. Fig. 3a and b present the *I*–*V* characterization of the devices based on the GeSe_2_ thickness-modulation (the corresponding optical images are shown in Fig. S4†). The different current densities are ascribed to the GeSe_2_ thickness and tunnel junction area. We calculated the current density (*J*) to normalize the influence of the tunnel area. For the case of the thinnest sample (9.8 nm), the device exhibited a high dark current. As we discussed above, DT is the dominant carrier transport mechanism under small *V*_ds_. The tunneling probability (*P*_TB_) is evaluated based on the equation^31^8
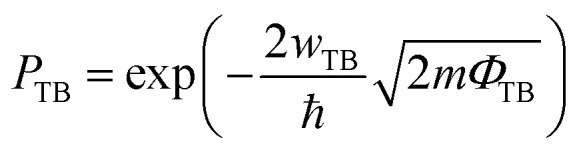
where ℏ is the reduced Planck's constant, *m* is the mass of the free carrier, and *w*_TB_ and *Φ*_TB_ are the width and height of the potential barrier. There is a bandgap difference of only 0.26 eV between the monolayer and bulk GeSe_2_.^17^ The tunneling probability can be suspected in terms of the barrier width, that is, the thickness. Therefore, the thinner GeSe_2_ vertical device shows a larger dark current.

Under 405 nm light illumination, all devices exhibit a positive photoresponse because the photon absorption generates electron–hole pairs in GeSe_2_ layers, which drift in opposite directions towards the graphene electrode. To evaluate the performance of photodetectors, several important parameters are explored quantitatively, including photo-to-dark-current ratio (PDCR), response (*R*_*λ*_), external quantum efficiency (EQE), and detectivity (D*). They are defined as^32–34^9
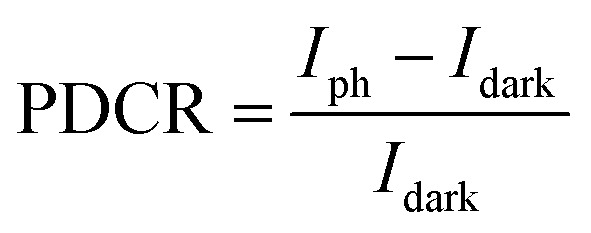

10 *R*_*λ*_ = *I*_ph_/*P*_in_*S*

11 EQE = *hcR*_*λ*_/*eλ*

12
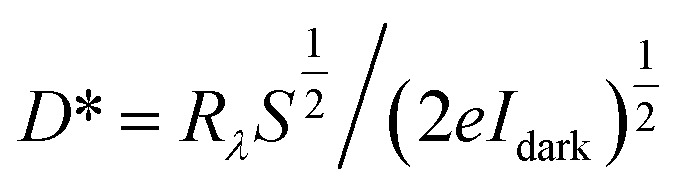
13
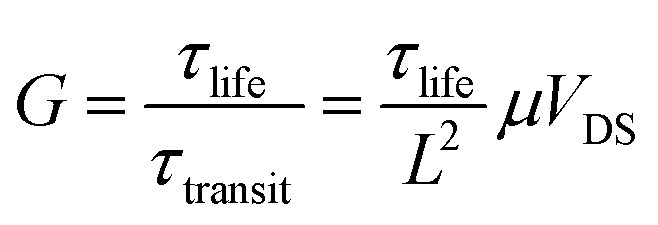
where *I*_ph_ is the photocurrent (*I*_illumination_ − *I*_dark_), *P*_in_ is the light power intensity, *S* is the area of the overlapped region, *h* is the Planck constant, *c* is the velocity of light, *λ* is the wavelength of exciting light, *L* is the length of the channel (GeSe_2_ thickness), and *μ* is the carrier mobility. The 26.8 nm GeSe_2_ device exhibits a better photoresponse with PDCR = 83.3, *R*_*λ*_ = 7.6*A*/*W*, EQE = ∼2316%, and D* = 5.0 × 10^10^ Jones. In general, a thicker sample has a higher absorption coefficient than a thinner one, producing a larger photocurrent. However, it has been reported that an increase in absorption layer thickness will result in a decrease in current gain. The reason is that increasing the thickness uniformly reduces the electric field intensity, resulting in significant reduction in the field-effect drifting of electrons and holes. Consequently, it is still essential to optimize the thickness of GeSe_2_ layers to function as light absorption and charge separation layers in the future.

**Fig. 4:**
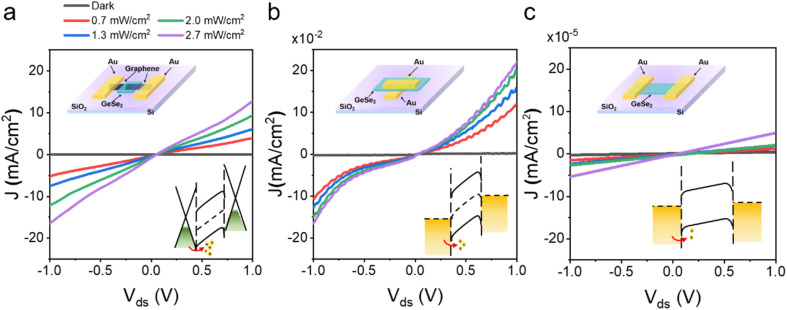
The electrical performance of three photodetectors with different architectures: (a) Gr/GeSe_2_/Gr (vertical), (b) Au/GeSe_2_/Au (vertical), and (c)Au/GeSe_2_/Au (horizontal), respectively. The inset diagram shows the structure schematic and band diagram.

To validate the superiority of this Gr/GeSe_2_/Gr vertical device, one set of comparisons between vertical and planar devices is shown in Fig. 4. The optoelectrical performance of vertical devices is much better than that of the planar device. The short transport channel results in a large electric field, leading to a highly efficient separation for the majority carriers and a high photocurrent. In addition, the vertical device with graphene electrodes shows better performance than that of the device with Au electrodes, which is ascribed to the UV light absorbance of Au electrodes reducing the carrier generation in the GeSe_2_ layer. Therefore, in Gr/GeSe_2_/Gr devices, the stronger input light and high carrier mobility of Gr layers lead to the devices showing excellent optoelectronic performance which is two orders of magnitude higher than those of the other architectures (see ESI Material Fig. S5† for details).

**Fig. 5:**
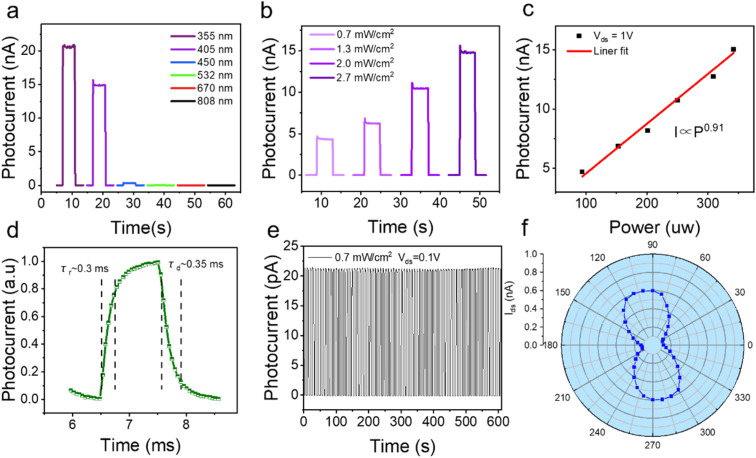
(a) Photocurrent–time (*I*–*t*) response at various wavelengths. (b) *I*–*t* response (fixed dc bias: 1 V) under a 405 nm laser with different power intensities. (c) Photocurrent as a function of light power density at *V*_ds_ = 1 V. (d) A typical photoresponse time curve for the rise time and the fall time. (e) Stability testing. (f) Anisotropic photoresponse under a 405 nm laser.

To demonstrate the spectrum-selective UV photodetection capability of our device, a series of photoelectric measurements were performed at different wavelengths. The time-resolved photoresponse at various wavelengths under the same light power (10 nW) is shown in Fig. 5a. These spectral photocurrent curves indicate an obvious suppression of device responsivity beyond the UV range. Fig. 5b shows the gradual increase in photocurrent with increasing illumination power density. Under an incident light of 2.7 mW cm^−2^, the *I*_light_/*I*_dark_ ratio has already reached over 1.12 × 10^5^. The photocurrent can be expressed by a power law relationship *I*_ph_ ∝ *P*^0.91^, as shown in Fig. 5c, suggesting efficient electron–hole pair generation and separation in the GeSe_2_ layers and Gr/GeSe_2_ Schottky interface.

The response speed is another pivotal parameter for PDs. We use the rise time (*τ*_r_) and decay time (*τ*_d_) to describe the response speed. The *τ*_r_ and *τ*_d_ are defined as the times of current changing between 10% and 90% of the maximum value, respectively. Importantly, the *τ*_r_ and *τ*_d_ are about 300 μs and 350 μs, which are measured using a home-made system with a voltage of 0.916 V. The system consists of a laser, an optical microscope, a current amplifier, and an oscilloscope. These values are smaller than those of lateral GeSe_2_ and Ga_2_O_3_ PDs. The ultrafast photoresponse of this photodetector is mainly attributed to a strong electric field at the junction between the Gr electrodes and GeSe_2_ layers, where carriers are spontaneously transferred. The anisotropy of the crystal structure and geometry of GeSe_2_ nanoflakes will directly lead to the anisotropy of photoelectric transport of carriers. Linearly polarized photodetection was investigated in Fig. 5f under 405 nm polarization illumination. It is clear that by rotating the polarization of the light, the photocurrent changes dramatically. These results further confirm that the photocurrent comes from the GeSe_2_ layer, which offers us a great opportunity to further exploit GeSe_2_ in the practical polarization-sensitive photodetector. Table 1 summarizes the performance of the Gr/GeSe_2_/Gr heterojunction photodetector and some reported photodetectors based on wide bandgap 2D nanomaterials for comparison. Notably, the performances of the Gr/GeSe_2_/Gr heterojunction photodetector in our work are better than those of the reported wide bandgap 2D nanomaterial-based photodetectors.

**Table 1:** Summary of 2D wide bandgap photodetectors

Materials	Measurement condition	*R* [mA W^−1^]	EQE [%]	D* [Jones]	Response time [ms]	On/off ratio	Ref
GeSe_2_	355 nm @ 1 V	3.7 × 10^4^	11 300	8.83 × 10^11^	0.3/0.35	>10^5^	This work
	405 nm @ 1 V	7.6 × 10^3^	2316	5.0 × 10^10^			
CdS	400 nm @ 2 V	0.18 × 10^3^	55.87	2.7 × 10^9^	14/8	10^3^	35
GaN	325 nm @ 2 V	1367	—	—	152.4/155.3	29.09	36
CaS	254 nm @ 2 V	4.2 × 10^3^	2050	10^13^	—	—	37
4H–SiC	310 nm @ 5 V	70	33	—	—	—	38
Ga_2_In_4_S_9_	360 nm @ 5 V	111.9 × 10^3^	3.85 × 10^4^	2.25 × 10^11^	40/50	2136	9
WS_2_	365 nm @ 5 V	53.3 × 10^3^	—	1.22 × 10^11^	30	2.94 × 10^4^	39
AlN	180 nm @ 5 V	—	—	—	110/80	10^2^	32
GQD	254 nm @ 5 V	2.1	—	9.59 × 10^11^	64/43	6000	40
ZnS	320 nm @ 5 V	120	50	—	—	—	41
CaTe	375 nm @ 5 V	30	—	—	54	—	42
SnS_2_	405 nm @ 5 V	0.695	0.22	7.39 × 10^6^	400	—	43
Diamond	210 nm @ 5 V	48	42	—	—	—	44
NiPS_3_	254 nm @10 V	126	—	1.22 × 10^12^	453/384	2 × 10^2^	10
BiOBr	315 nm @ 10 V	12739 × 10^3^	6.46 × 10^6^	8.37 × 10^12^	0.102	10^4^	12
CuGaS_2_	254 nm @ 10 V	5.1 × 10^3^	—	1.67 × 10^11^	1800/10 100	—	45
Ga_2_O_3_	250 nm @ 10 V	37	18	—	—	10^3^	6
TiO_2_	310 nm @ 10 V	3.27	—	—	—	—	4
ZnMgO	260 nm @ 10 V	1.664	—	—	—	—	46
GeSe_2_	266 nm @ 20 V	200	93	1.6 × 10^15^	300	100	17
h-BN	212 nm @ 20 V	0.1	—	2.4 × 10^8^	320/630	10^3^	13
CuBr	345 nm @ 30 V	3.17 × 10^3^	1126	1.4 × 10^11^	32/48	—	47

## Conclusion

In summary, we have fabricated a series of planar and vertical devices based on GeSe_2_ and Gr layers. Optical microscopy, AFM, Raman spectroscopy, and photoelectric measurements were systematically performed. The carrier transport mechanism of direct tunneling and thermoelectric emission was analyzed in the Gr/GeSe_2_/Gr device. Compared with the vertical Au/GeSe_2_/Au, horizontal Au/GeSe_2_/Au, and other 2D material photodetectors, the vertical Gr/GeSe_2_/Gr exhibits competitive high performance including an excellent responsivity of 37.1 A W^−1^, a maximum detectivity of 8.83 × 10^11^ Jones, a high on/off ratio (∼10^5^) at 355 nm, and a fast response time of ∼300 μs. We believe that the GeSe_2_-based vertical heterostructure will be a good candidate for application in UV photoelectronics.

## Conflicts of interest

There are no conflicts to declare.

## Supplementary Material

NA-004-D2NA00565D-s001
