# Supplementary material for: Two-dimensional wide-bandgap GeSe2 vertical ultraviolet photodetectors with high responsivity and ultrafast response speed
Source: Nanoscale Adv. 2022 Oct 3;4(24):5297–303. doi: 10.1039/d2na00565d (PMC9724610; doi:10.1039/d2na00565d)
Supplement: NA-004-D2NA00565D-s001 [file NA-004-D2NA00565D-s001.pdf]

Electronic Supplementary Material (ESI) for Nanoscale Advances.
This journal is © The Royal Society of Chemistry 2022

Supporting Information

Two-dimensional Wide-bandgap $GeSe_2$ Vertical Ultraviolet Photodetectors with High Responsivity and Ultrafast Response Speed

Yong Yan [a,*], Jie Li [a], Shasha Li [a], Mengna Wang [a], Gaoli Luo [a], Xiaohui Song [a], Suicai Zhang [a], Yurong Jiang [a], Ruiping Qin [a], and Congxin Xia [a,*]

a. School of Physics, Henan Normal University, Xinxiang, Henan province, China.

**E-mail: yanyong@htu.edu.cn and xiacongxin@htu.edu.cn*

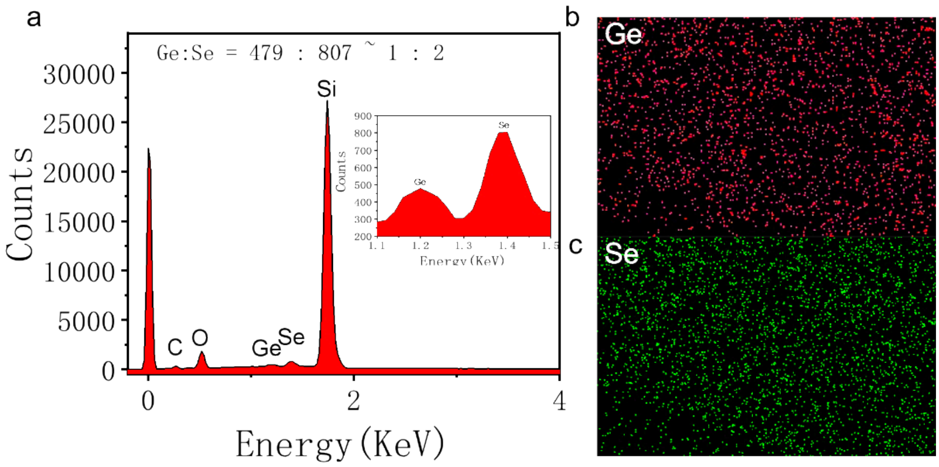

Figure S1. a) EDS spectrum of a graphene/$GeSe_2$/graphene. EDS elemental mappings of b) Ge and c) Se.

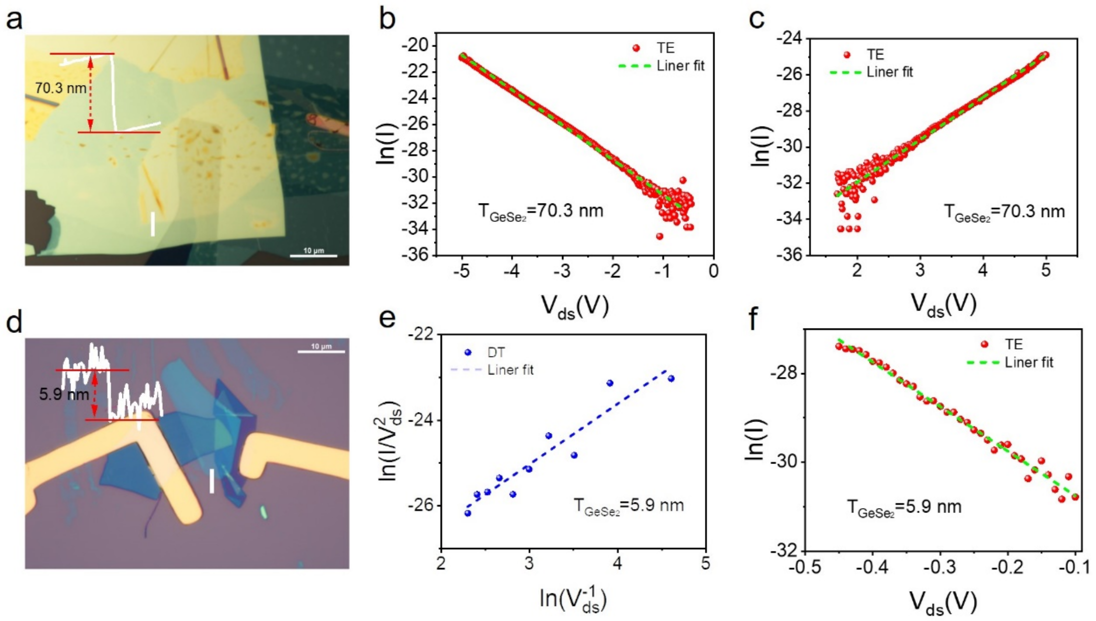

Figure S2.a) Optical micrograph of the graphene/$GeSe_2$(d=70.3 nm)/ graphene device and corresponding height profile. b) ln (I) vs. V_{ds} plot for the TE behavior at V_{ds} < -0.5 V and c) V_{ds} > 1.5 V. d) Optical micrograph of the graphene/$GeSe_2$(d=5.9 nm)/ graphene device and corresponding height profile. e) ln (I/V_{ds}^2) vs. ln (V_{ds}^{-1}) plot for the DT behavior at $|V_{ds}|$ < 0.1 V. f) ln (I) vs. V plot for the TE behavior between 0.1 and 0.5 V.

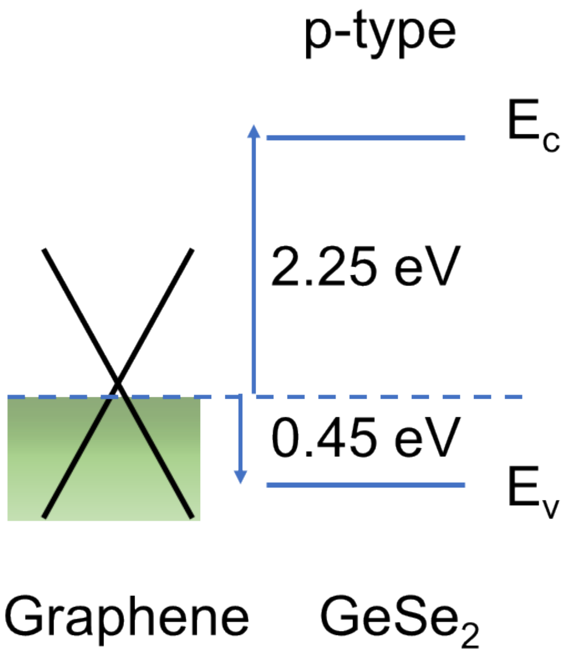

Figure S3. Band alignment of Gr/$GeSe_2$ heterostructures shows that the conduction

and valence band offsets are 2.25 eV and 0.45 eV.

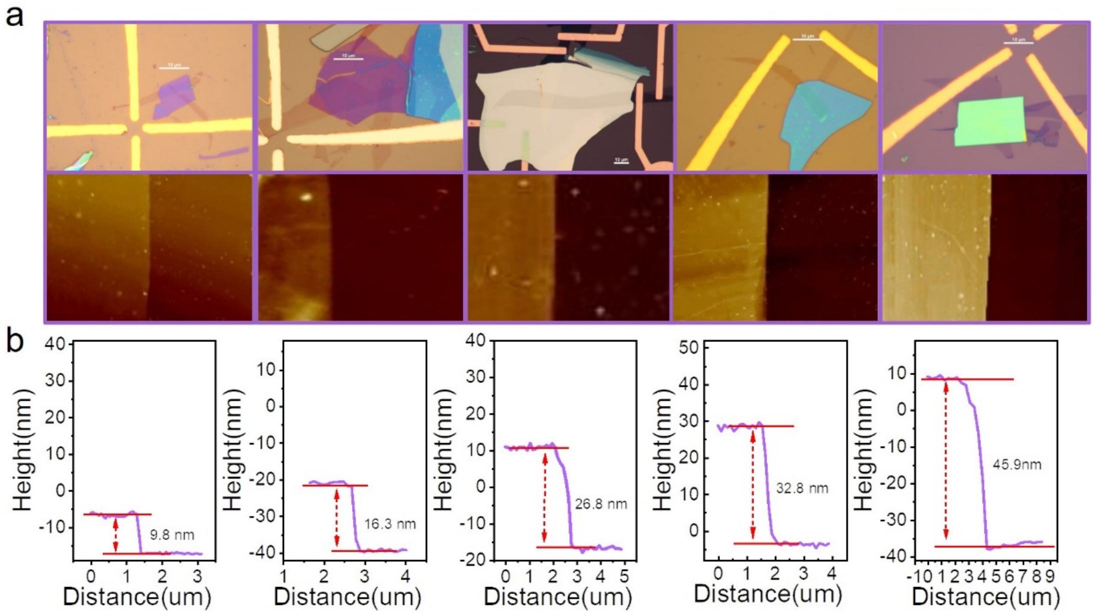

Figure S4. a) Optical micrograph of the heterojunction with different thicknesses and AFM images. b) Corresponding height profile. $GeSe_2$ thin layers with different thicknesses overlapping between graphene were identified by combining optical contrast and AFM measurements. The thickness of $GeSe_2$ ranged from 9.8 nm to 45.9 nm, and the effective area ranged from 2.9 to 98.5 um^2, depending on the specific device.

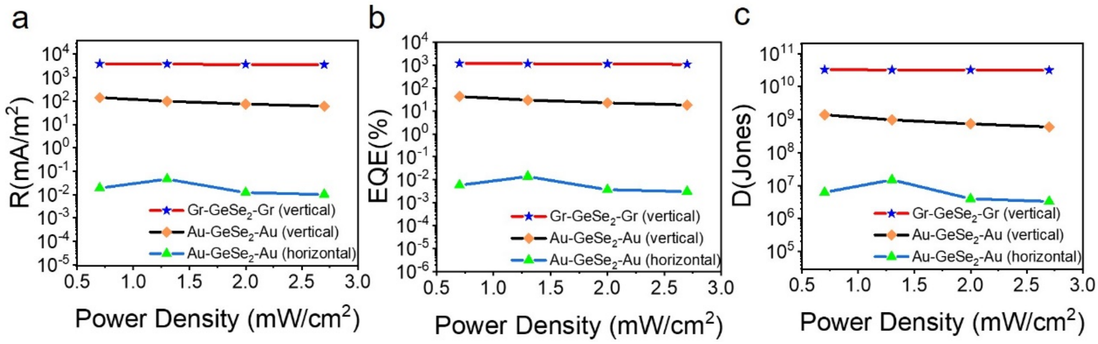

Figure S5. Optoelectronic properties of different heterogeneous structures. a) Responsivity (R), b) external quantum efficiency (EQE) and c) detectivity (D) under a 405 nm laser as a function of light power density under $V_{ds} = 1$ V.
